# Steel Strip Defect Sample Generation Method Based on Fusible Feature GAN Model under Few Samples

**DOI:** 10.3390/s23063216

**Published:** 2023-03-17

**Authors:** Cancan Yi, Qirui Chen, Biao Xu, Tao Huang

**Affiliations:** 1 Key Laboratory of Metallurgical Equipment and Control Technology (Wuhan University of Science and Technology), Ministry of Education, Wuhan 430081, China; 2 Hubei Key Laboratory of Mechanical Transmission and Manufacturing Engineering (Wuhan University of Science and Technology), Wuhan 430081, China; 3 Precision Manufacturing Institute (Wuhan University of Science and Technology), Wuhan 430081, China

**Keywords:** generative adversarial networks (GAN), deep learning, defect image generation, image cutting and stitching, strip steel surface defect, image classification

## Abstract

Due to the shortage of defect samples and the high cost of labelling during the process of hot-rolled strip production in the metallurgical industry, it is difficult to obtain a large quantity of defect data with diversity, which seriously affects the identification accuracy of different types of defects on the steel surface. To address the problem of insufficient defect sample data in the task of strip steel defect identification and classification, this paper proposes the Strip Steel Surface Defect-ConSinGAN (SDE-ConSinGAN) model for strip steel defect identification which is based on a single-image model trained by the generative adversarial network (GAN) and which builds a framework of image-feature cutting and splicing. The model aims to reduce training time by dynamically adjusting the number of iterations for different training stages. The detailed defect features of training samples are highlighted by introducing a new size-adjustment function and increasing the channel attention mechanism. In addition, real image features will be cut and synthesized to obtain new images with multiple defect features for training. The emergence of new images is able to richen generated samples. Eventually, the generated simulated samples can be directly used in deep-learning-based automatic classification of surface defects in cold-rolled thin strips. The experimental results show that, when SDE-ConSinGAN is used to enrich the image dataset, the generated defect images have higher quality and more diversity than the current methods do.

## 1. Introduction

Strip steel is one of the most important raw materials in the industries of automobiles, ships, and aerospace, and its quality directly affects the final performance of industrial products. During its production process, different defects, such as scratches and cracks, usually appear due to different processing techniques and rolling equipment [1,2]. The process that is prior to defect detection is classification, which aims to find the characteristics of each type of defects. In the past, detection and classification work were completed mainly by manual visual inspection. In other words, experienced workers judged the type of defects through human eyes to determine the fault type of the production equipment. However, this method has been gradually dropped mainly due to its vulnerability to subjective factors, low efficiency, and life safety risks [3].

Recently, artificial intelligent algorithms represented by deep learning have been widely applied in image classification and object detection [4,5,6,7,8]. Deep-learning-based classification models can classify objects without the extraction of features by humankind. Commonly, deep-learning classification models require a large number of samples [9]. However, it is more difficult to obtain strip steel defect samples than to obtain general data samples. There are many non-defective samples, but few defective samples in industrial production lines, so a defect dataset establishment becomes an urgent problem. Furthermore, the diversity of defect samples is insufficient, so it is impossible to reveal the complete defect distribution law [10,11]. Therefore, it is not easy to obtain abundant, diverse, and clear defect datasets in industries. In such a context, extending defect datasets through image generation has emerged as a reliable method, mainly by actively destroying defect-free workpieces or simulating defect images according to defect workpiece auxiliary drawing [12]. However, this traditional method is suitable only to simple defects. The defect images generated by auxiliary drawing are not natural enough, and there is still a gap between their texture characteristics and real ones.

The generative adversarial network (GAN) was first proposed by Goodfellow et al. [13] in 2014 and is currently one of the best image-generation models. Inspired by game theory, GANs consist of a generator and a discriminator. The generator captures the underlying distribution of real samples and generates new samples, while the discriminator is used to distinguish whether the input is a real sample or a generated one. The optimization process of GAN is a minimax game problem, and its ultimate goal is to achieve Nash equilibrium so that the generator can learn the probability distribution of real samples. Due to the advantages of GAN, many variant models have been developed so far. GAN generates images with higher speed, higher resolution, and better performance than other algorithms do. Therefore, GAN has potential to be tapped for industrial defect image generation [14]. It has achieved remarkable achievements in the generation of natural landscape images and face images [15]. However, there is still a long way to go before the application of GAN to surface defect training and the generation of strip steel in the metallurgical industry. The main reasons are summarized as follows. (1) GAN training requires a large number of samples, while the strip defect image sample itself is in deficiency, leading to a low generalization ability of the model [16]. (2) The diversity of generated defect images is insufficient. Strip defect images are divided into different types according to their defect characteristics, such as shape defect, location defect, distribution defect, and color defect. Consequently, the fewer the available samples are, the less diverse the generated samples are [17].

In the context of sample shortage, it is feasible to obtain similar images of a certain type of samples by enriching them through a small amount of training. The recently proposed SinGAN [18] introduces a GAN method, which is able to accept the training of unconditional generation and coordination of a single image and generate real images from low-to-high resolution stage by stage. Later, Tobias Hinz [19] proposed ConSinGAN based on SinGAN. It trains multiple stages with different learning rates simultaneously and does not generate images in the intermediate stage but from end-to-end. All of these steps have rendered the generated images more realistic and less time-consuming. It has been found that there are many training stages in SinGAN, leading to low training efficiency. However, if the stages are reduced, the generated images are not full in the global picture. ConSinGAN adopts multi-stage training for better speed, but new problems, such as image overfitting and frame, emerge. The above two methods do not give more weights to images with larger resolution during the training process, and the number of iterations is fixed, so they work well in completing image-generation tasks, such as house buildings and natural landscapes. However, it does not work well in strip defect image generation because such problems as insufficient diversity and serious overfitting appear. To sum up, traditional generative models require a large number of defect samples as a dataset, and strip defect samples are few, so they cannot be well trained. However, the current single-sample and few-sample generation model works well only on natural images and is not suitable for the generation of steel strip defect samples. Consequently, a defect generation method named Strip Steel Surface Defect ConSinGAN (SDE-ConSinGAN) has been proposed by this paper on the basis of single-image training through a generative model. The presented SDE-ConSinGAN aims to learn more texture details and colors of defective images. In addition, features of the real samples will be fused as new training samples to increase the diversity of generated images. Finally, the defect dataset obtained by the proposed SDE-ConSinGAN model will be used for classification. The experimental results demonstrated that the proposed SDE-ConSinGAN works well in the generation, classification, and subsequent processing of defect samples.

In summary, the main contributions of this article are as follows:

(1) By introducing a curve size-adjustment function and a dynamic adjustment function for the number of iterations, combined with the ECA (Efficient Channel Attention) attention mechanism, a new generation method called SDE-ConSinGAN is proposed to economically generate images with high quality and a variety of flawed images.
(2) An improved image-cutting and fusion method has been proposed. It can cut and fuse multiple images of the same defect type but different defect characteristics, and then, the images can be treated as a new sample for GAN training for more diversity.
(3) SDE-ConSinGAN is an enhancement method for strip defect datasets; training focuses on the texture details of defect images, guides the generator to learn more noteworthy features, and speeds up training to obtain the best performance for different types of defect images and task results. Experimental results show that defect classification models trained with SDE-ConSinGAN-augmented datasets perform significantly better than datasets composed of other generative models.

## 2. Related Work

This section will discuss related work on surface defect classification, image-generation, and fusion methods.

### 2.1. Surface Defect Classification

Undoubtedly, deep learning is a fine and powerful subfield of machine learning, and its core idea is to automatically learn the hierarchical feature representation from a large number of images through the convolutional neural network (CNN). Recently, it has been widely used in image identification [20]. Liu et al. proposed an algorithm based on CNN by using a structure of two-layer convolution with a max-pooling layer for defect classification [21]. Faghih-Roohi et al. increased convolutional and pooling layers to three layers, bringing about higher accuracy [22]. SHANG L et al. adopted a CNN on the basis of the Inception-v3 structure to distinguish normal and defective images, consequently achieving the desired results [23]. However, the methods described above and the current mainstream deep-learning classification methods require a large number of data samples. When the number of data samples is few, it will be hard to obtain sample data, consequently having a bad impact on the classification ability of the model [24,25,26]. Therefore, it is necessary to develop a surface defect generation method with few samples to achieve their inspection and classification.

### 2.2. Image-Generation Method

Traditional image generation is to expand the dataset by transforming the original image to generate a new image, which includes mainly the following methods. (1) Physical transformation. That is, new images are generated by turning the image horizontally or vertically, scaling it according to a certain proportion, clipping it, translating it, copying it, and utilizing other means of changing the shape, size, and position of the image. This kind of method is simple, efficient, and suitable for simple image expansion tasks. (2) Interpolation method. During rotation, translation, or reduction in an image without a solid color background, and if maintaining the size of the original picture is necessary, the general approach should be the interpolation method to fill the image of unknown pixels, such as constant filling, edge filling, reflection filling, symmetric filling, and so on. The interpolation method transforms the content of the image to a certain extent and improves the image expansion greatly. (3) Noise transformation. It is performed by adding or removing some noise on the image or changing the attributes to generate a new image, such as by increasing Gaussian noise or changing the brightness, saturation, contrast, and other parameters of the image [27]. This kind of method can obviously bring about a difference from the original image visually, but it does not change the essential content of the image. The method, which is based on deep-learning, can obtain deep and dataset-specific feature representation through the learning of a large number of samples, which can express the dataset more efficiently and accurately, extract more robust abstract features, and have better generalization ability [28]. Generative models based on deep learning are divided mainly into two categories. The first category is models which can fully describe sample characteristics and solve parameters via maximum likelihood estimation, such as the generative model GM [29], autoencoder AE [30], variational autoencoder VAE [31], etc. Lee et al. proposed a two-stage framework composed of RQ-VAE and RQ-Transformer to efficiently generate high-resolution images [32]. The second category is models which can generate samples from unknown data of distribution functions by means of learning, such as the Artificial Neural Network (ANN) [33] and the Deep Belief Network (DBN) [34]. On the basis of ANN, Tan et al. [35] mapped coarse fields to fine fields for the generation of fine-scale training samples through a mapping network. However, an accurate obtainment of distribution function is not easy in the real world. Hence, the second type is usually used for sample generation, but there are still problems with it, such as diversity deficiency and poor quality [36,37]. These problems will seriously reduce its training efficiency and accuracy.

GAN makes full use of the confrontation process between the generator and discriminator for training. As more and more scholars pay attention to the GAN model, plenty of variant models are derived to meet the realistic need [38,39,40]. It was for the first time that the Deep Convolutional Generative Adversarial Neural Network (DCGAN) [41] introduced the CNN idea into GAN. It aims to generate samples by adopting the design idea of “convolution with upsampling” and making use of the reverse CNN of DCGAN. Mehdi Mirza et al. [42] proposed Conditional Generative Adversarial Neural Networks (CGANs), which add conditional information to traditional GANs to control the training process of the generator and discriminator, consequently improving the stability of model training. CycleGAN [43] has been put forward to address the unavailability of paired training samples. It aims to generate complex samples by mapping images from source domain to the target one. The above methods use GAN and its variants to enrich samples. However, they are not suitable to industrial defect image generation due to their sample defect and diversity insufficiency. Currently, there are only a few methods which can train GAN models with a few samples or even on a single image, and there are only a few models which can be trained on a single “natural” image [44,45]. In the task of generating a single strip defect image, it cannot obtain good results.

### 2.3. Image-Fusion Method

Many image-fusion methods have been used to fuse multiple images with different features into one image. The simplest image-fusion method is to directly perform pixel weighted superposition [46,47], which can visualize image overlapping directly. However, it is not suitable for defect image fusion. OpenCV2 [48] based on the Speeded Up Robust Features (SURF) can achieve seamless splicing and fusion of two images, provided that the two images have similar areas. Obviously, it is not suitable for defective images with high structural uncertainty. NVlabs came up with a style transfer StyleGAN [49,50] in 2019. Through the method of feature interpolation, the latent noise in the training layer can be smoothly fused to achieve seamless mixing of two images. Nevertheless, StyleGAN also belongs to one type of GAN, which requires a large number of samples for training to achieve image mixing task. Actually, StyleGAN does not work well for defective datasets with few samples.

## 3. Materials and Methods

**Figure 1:**
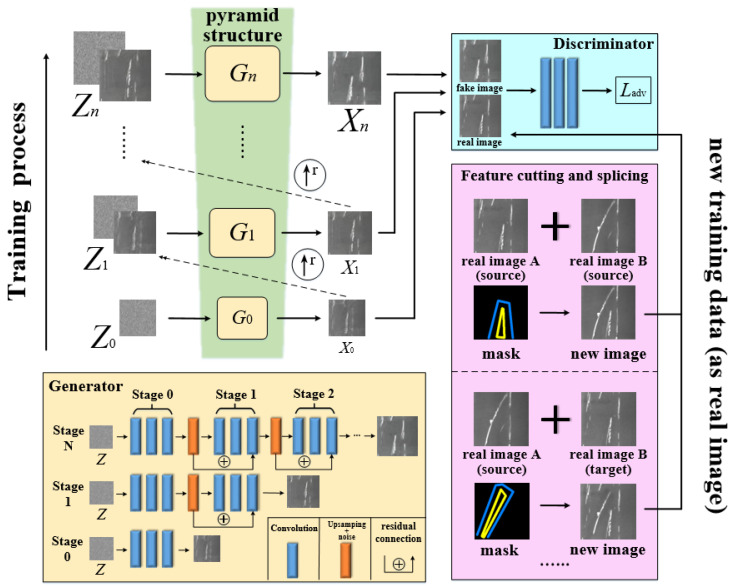
The Framework for Image Generation and Feature Fusion for Strip Defects.

This section will discuss the proposed SDE-ConSinGAN in detail. In order to generate more realistic images, a sample generation and feature-fusion framework is constructed through SDE-ConSinGAN and Graphcut image-cutting methods. As is shown in Figure 1, SDE-ConSinGAN and Graphcut are divided into two parts. In the first part, the SDE-ConSinGAN model is used for training, and it can generate samples that are similar to original defect images. In the second part, the improved Graphcut is employed to cut and fuse two images with different detailed features but from the same defect type to obtain a new defect image with multiple features. Then, the new images will be used for training and generating more diverse samples.

### 3.1. High-Quality Defect Image Generation Based on SDE-ConSinGAN

The SDE-ConSinGAN model is designed according to the pyramid structure that is used in SinGAN. The image resolution is divided into multiple stages, namely, from low resolution to high resolution. Training and generation are also completed from low resolution to high resolution. At each stage, the generator learns features and generates image samples. The region of the processed image is called the receptive field. Then, the generated image is applied to the discriminator for identification. When the discriminator cannot distinguish true or false images from the current training samples, the next stage of training will begin. All the G and D have the same network structures, which consist of several groups of convolutions (with a size of 3 × 3) and a receptive field (with a size of 11 × 11). The size of the receptive field is equal to that of the convolution kernel.

**Figure 2:**
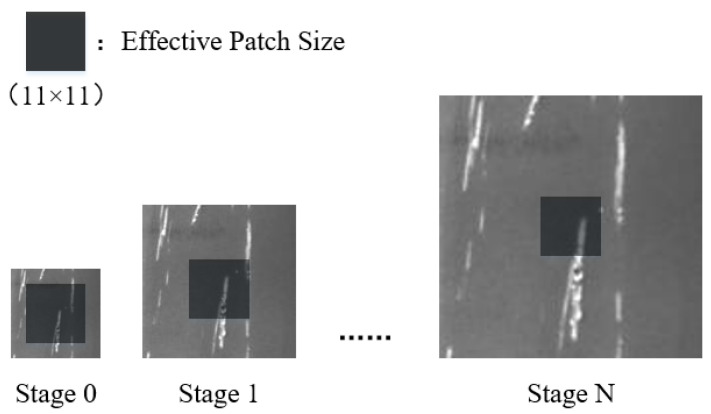
Receptive fields of images with different sizes.

As is exhibited in Figure 2, since the size of the receptive field is fixed, the image will be the smallest when the resolution is the lowest. Even if the receptive field is small, it can cover most of the image areas. With the increase in training stages, the input image gradually becomes larger, and the range covered by the receptive field becomes smaller and smaller. In other words, the image area that is processed each time gradually decreases. Each time the discriminator is employed for discrimination, it splits the original image into many small receptive fields and judges whether the local image in the generated image is a real image or not. This allows the generator to generate images that are different in the overall picture but very similar in each receptive field, contributing to a Nash equilibrium between the generator and discriminator.

The multi-scale architecture used in ConSinGAN is an efficient way to implement the pyramid model. The image generation starts from the scale with the lowest resolution and goes up step by step. Noise adding, which is used for diversity increase, will not stop until the generator reaches the scale with the highest resolution. In the $G_{0}$ stage, the Gaussian noise image $Z_{0}$ is imported into the generator as the original sample, as is shown in (1):

(1) $$X_{0}=G_{0}\left(Z_{0}\right)$$

In the early training stage, the effective receptive field usually occupies a large proportion of one image, so its overall structural layout can be learned. As the training progresses, the image resolution gradually increases, and each generator $G_{n}$ can learn the details that another generator failed to learn in the previous stage. Apart from accepting a sampled image generated by the previous generator, each generator adds additional noise, as shown in (2):

(2) $$X_{n}=G_{n}\left(Z_{n}+\left(X_{n-1}\right){↑}^{r}\right)\;,\;n<N$$

where the symbol ${↑}^{r}$ is an up-sampling image of image $X_{n-1}$.

**Figure 3:**
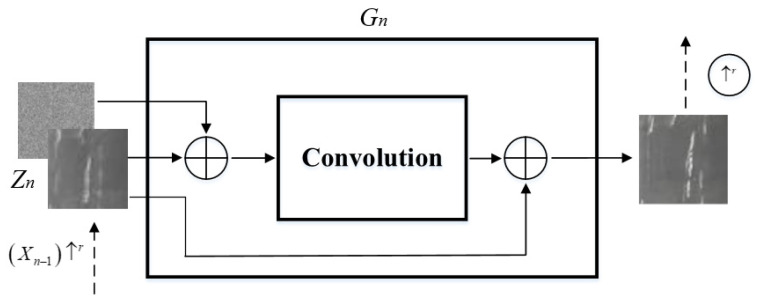
The structure of the generator.

As shown in Figure 3, all generators have a similar structure and function. In other words, they can send the mixed extra Gaussian noise *Z* and $\left(X_{n-1}\right){↑}^{r}$ added at each stage to the generator to ensure that the added noise can retain some weights during the process of generating images. The convolutional layer functions as a supplement to the missing details in $\left(X_{n-1}\right){↑}^{r}$. Then, (2) can be transformed into the following (3):

(3) $$X_{n}=\left(X_{n-1}\right){↑}^{r}+conv_{n}\left(Z_{n}+\left(X_{n-1}\right){↑}^{r}\right)\;,\;n<N$$

where the symbol $conv_{n}$ refers to the convolution operation, which consists of three identical convolution blocks. Each one is composed of a convolutional layer (with a convolution kernel of 3 × 3), a normalization layer, and an activation function layer.

The discriminator consists of five simple convolution layers. After the image distribution at the current scale is obtained through the sampling method, the image data obtained from the last stage and current stage will be input into the discriminator to calculate the loss function.

#### 3.1.1. Image Size Adjustment

As is mentioned above, the model in the early training stage focuses mainly on the learning of the overall structural layout of images. In the later training stage, it focuses on the learning of texture details of images. Due to the great uncertainty of the structure, texture details and defect color are taken mainly as standards of defect classification. Therefore, the texture and details should be considered, and the structure and layout should be less focused on. In the process of training, the model needs to use multiple reconstructed images with different resolutions. The number of images with different resolutions depends on the number of stages during the process of training. The generation of natural landscape samples in ConSinGAN requires a specific global image layout and learning in low-resolution stages. As defect images require more high-resolution stage learning, we design a curve function to adjust the image size scaling, as is shown in (4):

(4) $$x_{n}=1-x_{N}r^{\left[\left(1+n\right)/\mathrm{lg}\left(N\right)\right]\mathrm{lg}\left(1+n\right)+1}$$

where the value range of *n* is $\left[0,N-1\right]$. *r* is an artificially set scaling scalar, the default of which is set to be 0.55. Compared with ConSinGAN, the density of the high-resolution stage is higher than that of the low-resolution stage when the curve function of (4) is adopted for size adjustment. For example, as shown in Table 1, when the number of training stages is 6, it is found that the new size-scaling method has more high-resolution stages (the real sample image size is 200 × 200) than the original one does.

The resolutions of all other stages are larger than those of the original method, except the maximum resolution, which is equal to the size of the real image. In addition, in (4), with the increase in stage numbers, the scale of the image size is basically unchanged. In other words, in the first few training stages, low-resolution stage learning is still necessary. The stage from low resolution to high resolution is a gradually approaching process without a large span. Its advantage lies in the partial retention of the image global structure, which can guarantee that the generated image will not completely deviate from the original image features.

#### 3.1.2. Loss Function

**Figure 4:**
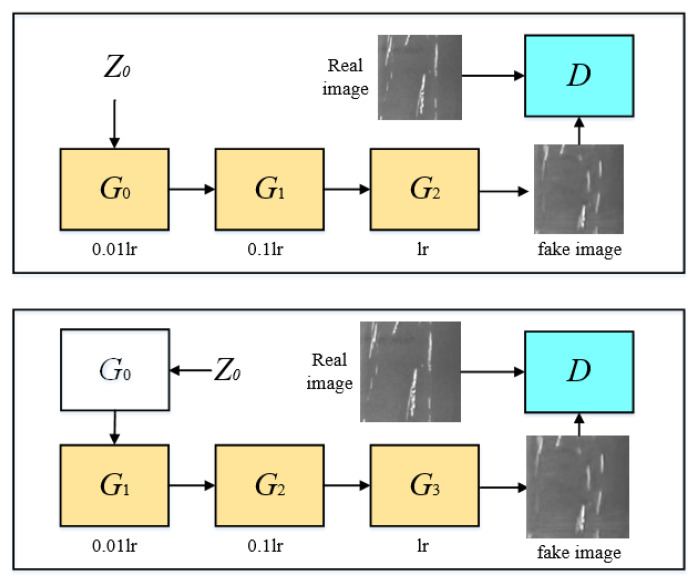
Multi-stage training structure with different learning rates.

The key to single-image generation is the multi-scale, multi-stage training method [19]. As is exhibited in Figure 4, the generators of the current stage and the first two stages are selected to jointly train and update parameters by default, and the parameters of all other generators are frozen. Among them, the parameter *lr* refers to the learning rate, which is a hyperparameter that is artificially set in the experiment. The higher the value is, the more faithful the features are to the original images. Three generators, $G_{0},G_{1},G_{2}$, are trained at the same time, and the learning rates of $G_{0},G_{1}$ are adjusted to 1/100 and 1/10. When the training of $G_{0},G_{1},G_{2}$ is completed, the parameters of $G_{0}$ will be fixed. Then, the three generators $G_{1},G_{2},G_{3}$ are trained at the same time, and the learning rates of $G_{1},G_{2}$ are adjusted to 1/100 and 1/10. Similarly, the same practice will go on.

Sequential training will be performed from the lowest resolution scale to the highest one. When three generators or above are trained simultaneously, only the parameters of the latest three generators will be updated, and other generators will be fixed. In the n-th stage, the loss function of GAN contains adversarial and reconstruction loss. The loss function of GAN is exhibited in (5):

(5) $$\underset{G_{n}}{\min}\underset{D_{n}}{\max}L_{adv}\left(G_{n},D_{n}\right)+\alpha L_{rec}\left(G_{n}\right)$$

where $L_{adv}$ is the adversarial loss, $L_{rec}$ is the reconstruction loss, and α is the artificially set weight. The adversarial loss refers to the gap between the patch distribution of the generated images and that of the real images. The reconstruction loss is used to determine noise that can generate an image. The former employs the Wasserstein Generative Adversarial Neural Networks with Gradient Penalty (WGAN-GP) [51] loss, a truncation-pruning method for higher quality generation results. The latter is used to improve the training stability, and it is optimized in this article. When the value of n is zero, replace $x_{0}$ with a fixed noise $Z_{0}$, as is shown in (6):

(6) $$L_{rec}=\left\{\begin{array}{l} {\left\|G_{n}\left(x_{0}\right)-x_{n}\right\|}_{2}^{2}\;,\;n<N\\ {\left\|G_{n}\left(Z_{0}\right)-x_{n}\right\|}_{2}^{2}\;,\;n=0 \end{array}\right.$$

The training method of the discriminator remains the same. Its input is generated images or real images, and its training goal is maximization.

#### 3.1.3. Attention Mechanism

Theoretically, convolutional feature extraction is a point that is worthy of research. There are two main attention mechanisms commonly used in convolutional neural networks, such as channel attention and spatial attention, which can correct features. Channel attention will determine which feature channel is more important through modeling and then enhance or suppress different channels according to different tasks. It acts on the perceptual intensity of each position in the input image. In other words, it will pay much more attention to the defect or foreground area of the images. Spatial attention focuses on where the information part is located. In most cases, the region of interest is only a small part of the images, so the essence of spatial attention is to locate the target and carry out some transformation or obtain weight. The attention features obtained by spatial attention and channel attention are complementary [52]. The ConSinGAN network assigns the same weight to each channel when extracting features. For the defect image generation task, it is critical to automatically obtain weights of each feature channel through learning, strengthen useful features, and weaken useless ones according to their weights. However, for complex and irregular strip steel surface defect images, we do not pay attention to the location of defects. Spatial attention has the feature of learning global arrangement, which is contrary to our previous idea. Therefore, only channel attention is introduced in this paper. In this way, important features are enhanced, and unimportant ones are weakened, leaving extracted features more directional.

**Figure 5:**
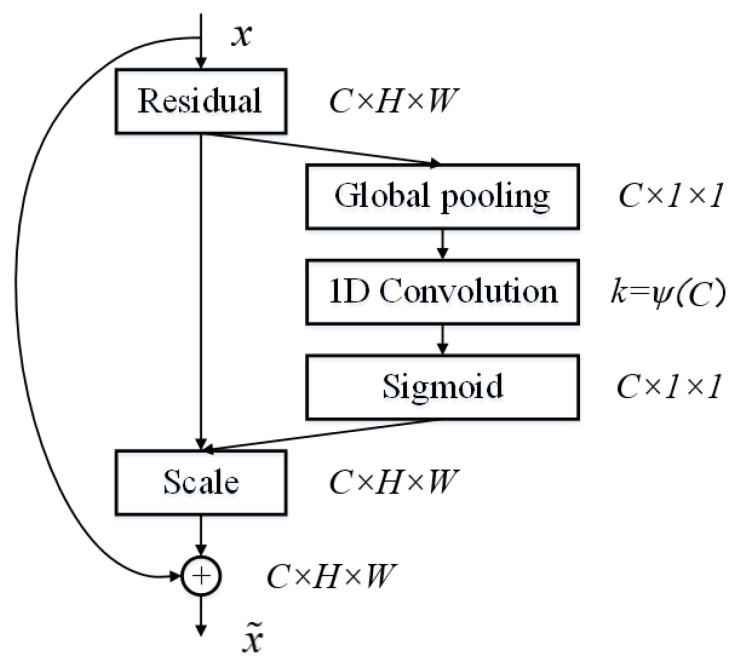
ECA-Net attention mechanism module.

Improved Channel Attention ECA-Net [53] is an improved version of SE-Net [54]. It can be easily embedded into most CNNs no matter how different these CNNs are in levels, depths, and stages. SE-Net uses global average pooling for each channel separately and then uses two nonlinear FC layers and a Sigmoid function to generate channel weights. The two FC layers are designed to capture nonlinear cross-channel interactions and render the model less complicated through dimensionality reduction. However, the measure of dimensionality reduction affects the weight learning of channel attention. In order to solve this problem, ECA-NET is proposed for improvement. As is illustrated in Figure 5, when the method SE-Net is adopted, the FC layer brings about side effects to attention prediction due to its dimensionality reduction operations, while ECA-Net can successfully refrain from doing so. Channel attention is generated by fast 1D convolution, the kernel size of which can be adaptively determined by nonlinear mapping of channel dimensions.

ECA-Net has been proven to be more effective than other channel attention models. Importantly, ECA-Net is a lightweight model with no additional parameters and can be easily embedded into other frameworks. When the ECA-Net module is embedded into the convolution operation conv of (3), it can improve the performance of CNN and enhance the accurate learning ability of the generators with regard to image defects without increasing the amount of computing time. These two steps can improve the generated image quality.

**Figure 6:**
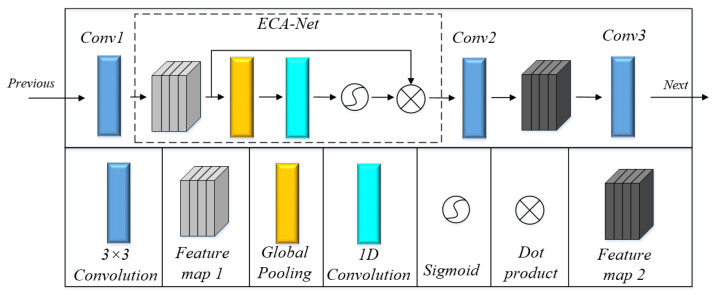
ECA-Net embedding position.

In Figure 6, the generator consists of three convolutional layers (Conv1, Conv2, and Conv3). It is the best choice to embed the ECA-Net between *Conv*1 and *Conv*2 because of upsampling, filling operations, and additional noise adding between Conv2 and Conv3. Upsampling and filling will increase the computation of the ECA-Net module, and noise will interfere with the ECA’s ability to distinguish effective features from the background. Therefore, it is the optimal choice to place ECA between Conv1 and Conv2, which not only achieves the desired results of attention mechanism but also avoids the noise interference.

#### 3.1.4. Iteration Times Variation Function

Although ConSinGAN trains multiple stages at the same time, it still takes a long time when faced with repeated training tasks. In ConSinGAN, the iteration time in each stage is 2000 by default. It is found that the greater the iteration time in the low-resolution stage is, the more alike the generated image structures are to those of the original images, and the more similar their details are. If iteration time in the low-resolution stage is set to be 2000, only slight disturbance will appear in the generated defect images, which results in overfitting. In the above study, the focus of model learning is placed on the high-resolution stage. On this basis, iteration times in the low-resolution stage are reduced, which not only shortens model training time but also places the focus on defect detail learning. (7) shows the iteration times variation function:

(7) $$iter=\frac{niter}{2}\left(1+\frac{\nu_{\max}}{v_{ave}}\mathrm{lg}\left(\frac{N+1}{N-n+1}\right)\right)$$

where the symbol *iter* is the iteration times required for training in the current stage; *niter* is the number of custom iterations; $\nu_{\max}$ is the rate where training peaks; $\nu_{ave}$ is the average training rate; *N* is the total number of training stages; and *n* is the number of current training stages. Even if the iteration times in some stages are more than those of the original stage, the iteration time is reduced in an overall picture, which can shorten training time to a certain extent.

### 3.2. Image-Feature Fusion Based on Graphcut

During the procedure of strip steel production and processing, its surface defect types and severity are different due to working environment differences, thus leading to the obtainment of different defect images. Since ConSinGAN and SinGAN are generated based on a single image, the image content generated by them is highly limited to the semantic information provided by the training image. In other words, it is less “creative”. New samples can be obtained for training through the image-feature fusion method. Image cutting and fusion is a method which can splice a certain feature of the A-type image into the B-type through a seam mask when the B-type does not have what the A-type has. In this way, the generated new image has both partial features of the two types. The SDE-ConSinGAN model is used to generate new defect images by changing part of the image structure and texture features. Therefore, the feature-fusion function of Graphcut plays an important role in the generation task of the SDE-ConSinGAN model.

There are two steps of the Graphcut method for image cutting and splicing. First, select a partial image in the original image and place it on the output image. Second, find the pixel gap between the local image block and the existing pixels in the original output image. Moreover, determine which one among the output images really becomes the pixels in the local image block and which one remains unchanged [55].

**Figure 7:**
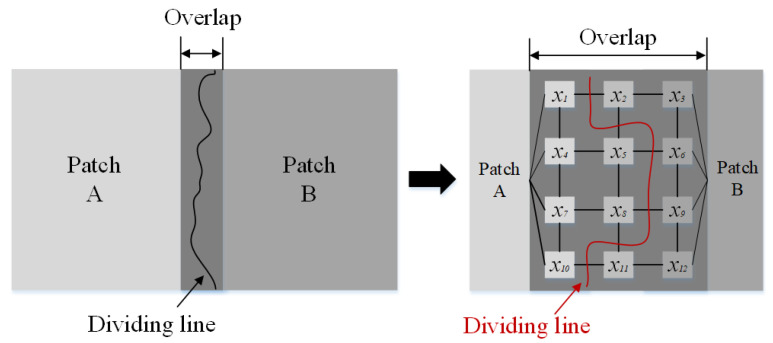
Graphcut method for image cutting.

As is illustrated in Figure 7, when two patches (Patch A and Patch B) are placed together, there will be a certain overlapping. In order to render the gap between the two as inconspicuous as possible, it is necessary to create a dividing line. In the left of the line, Patch A mainly contributes to the image pixels, and Patch B is on the other side. In Figure 7, the node where each x is located is actually a pixel in the overlapping area, and each connection between nodes has a cost. Our purpose is to use a dividing line to cut the node connection and minimize the cost of the connection cut. Each x may come from either *A* or *B*. Assuming that there are two adjacent output pixels $x_{1}$ and $x_{2}$ in the overlapping area, the symbol $A\left(x_{1}\right)$ and $B\left(x_{1}\right)$ are used to represent the grayscale of $x_{1}$ point in image *A* and image *B*; the symbols $A\left(x_{2}\right)$, $B\left(x_{2}\right)$ are used to represent the grayscale of the $x_{2}$ point in the image *A* and the image *B*. Therefore, the connection cost between points $x_{1}$ and $x_{2}$ is defined as follows:

(8) $$M\left(x_{1},x_{2},A,B\right)=\left\|A\left(x_{1}\right)-B\left(x_{1}\right)\right\|+\left\|A\left(x_{2}\right)-B\left(x_{2}\right)\right\|$$

The only action that needs to be performed is to find a cutting seam and minimize it. For $x_{1}$ and $x_{2}$ in all cutting seams, the sum of (8) is the minimum. When this seam is found, the pixels on the left are copied from *A*, and those on the right are copied from *B*. The Graphcut method is highly creative, easy to operate, and highly adaptative because it is free from the training process that other feature-fusion methods have.

Although the Graphcut method can bring sound seam around a given texture, it still produces visible artifacts if there is no good seam at that point, which is especially serious for strip defect images. These imperfections can be hidden through adjusting the pixel values on the seam. After the Graphcut method is used for image-feature splicing, the fade-in and fade-out weighted-average method is adopted for fusing the seams. The basic idea of this method is to replace the pixel value with the gray-scale weighted value of the pixels in the overlapping area [56]. After many experiments, it can achieve a sound effect of eliminating seam marks.

First, the weight requires calculating. The distance from pixel $\left(x,y\right)$ to the boundary of mask area (overlapping area) is set as *width*, namely $\varpi_{i}=1/width,i=1,2$. $\varpi_{1}$ and $\varpi_{2}$ respectively represent local image weights on both sides of the seam, and they satisfy the condition of $\varpi_{1}+\varpi_{2}=1$ and $0<\varpi_{1}<1\;,\;0<\varpi_{2}<1$.

The symbols $I_{1}\left(x,y\right)$ and $I_{2}\left(x,y\right)$ are pixel gray values on both sides of the seam point $\left(x,y\right)$ before fusion. The symbol $I\left(x,y\right)$ is the pixel gray value after fusion at point $\left(x,y\right)$. Their calculation formula is as follows:

(9) $$I\left(x,y\right)=\left\{\begin{array}{ll} I\left(x,y\right) & \left(x,y\right)\in I_{1}\\ \varpi_{1}I_{1}\left(x,y\right)+\varpi_{2}I_{2}\left(x,y\right) & \left(x,y\right)\in I_{1}\cap I_{2}\\ I\left(x,y\right) & \left(x,y\right)\in I_{2} \end{array}\right.$$

**Figure 8:**
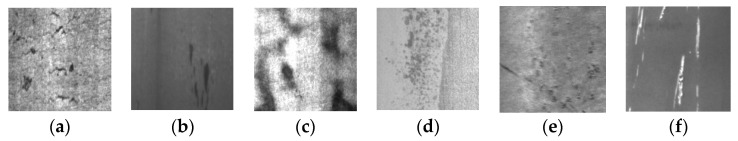
Surface defect sample of original strip. (**a**) Cr. (**b**) In. (**c**) Pa. (**d**) PS. (**e**) RS. (**f**) Sc.

**Figure 9:**
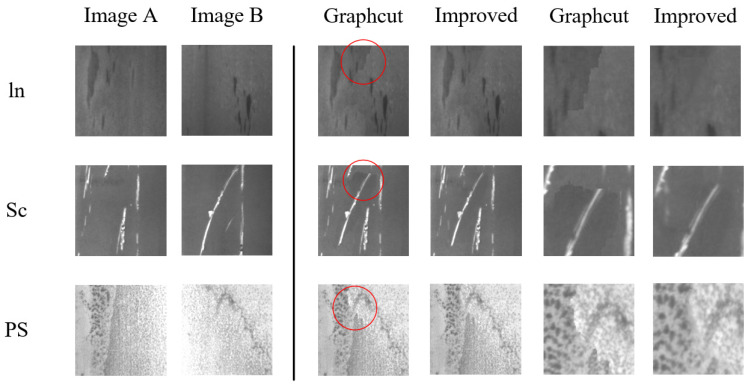
The improved Graphcut method for image cutting.

The defect images in this paper come from the NEU strip surface defect dataset provided by Song et al., which consists of six types of defects [57]. That is, crazing (Cr), inclusion (In), patches (Pa), pitted surface (PS), rolled-in scale (RS), and scratches (SC) defects, as is illustrated in Figure 8. The splicing traces can be eliminated when the above method is adopted for pixel grayscale fusion, and its effect is shown in Figure 9.

Figure 9 lists the results of feature fusion of In, PS and Sc defects through the im-proved Graphcut method. The last two columns display partially enlarged images which are circled in red. It can be seen that there are still tiny marks left when the original Graphcut method is employed for cutting and splicing. However, this problem can be solved when the pixel grayscale fusion method is added.

## 4. Experiment and Results

In this paper, the experiment focused on defect image generation of strip steel with a few samples. The experiment revealed that the SDE-ConSinGAN model is valid, and its generation results and performance were compared with those of others. The results showed that the SDE-ConSinGAN can be used for defect image generation and defect testing of a strip steel surface.

### 4.1. Dataset

The defect images in this paper come from the NEU strip surface defect dataset shown in Figure 8. Since our model is trained with minimal sample data, only 10 grayscale images (both in size 200 pixel × 200 pixel) from each defect type are selected as the original defect images. All experiments are conducted on the same server with the following performance configuration: i5-11400F CPU, 16Gddr4 memory, Nvidia RTX3070 GPU with 8 Gb memory, and the Windows 10 operating system.

### 4.2. Single-Image Generation

**Figure 10:**
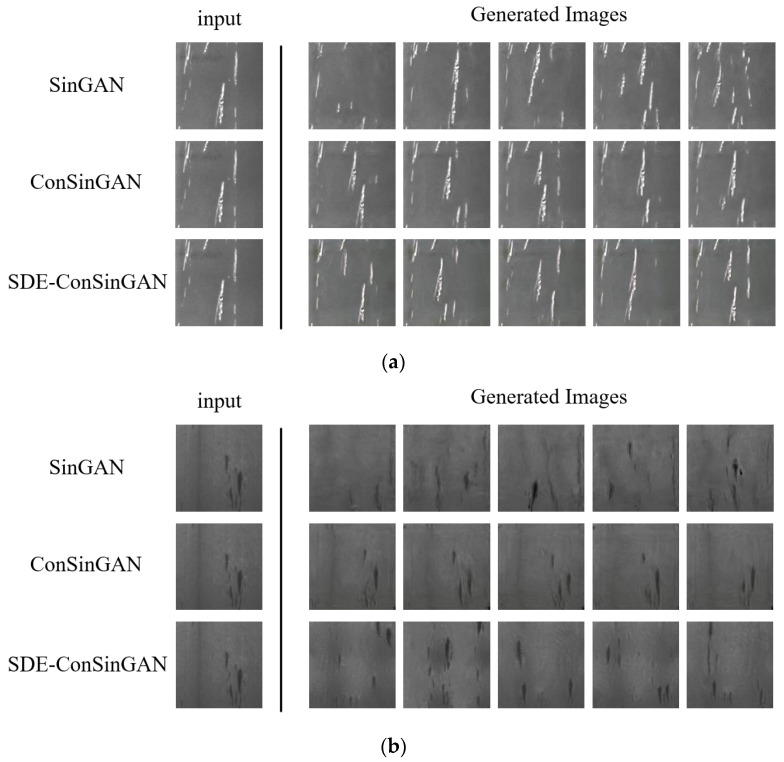

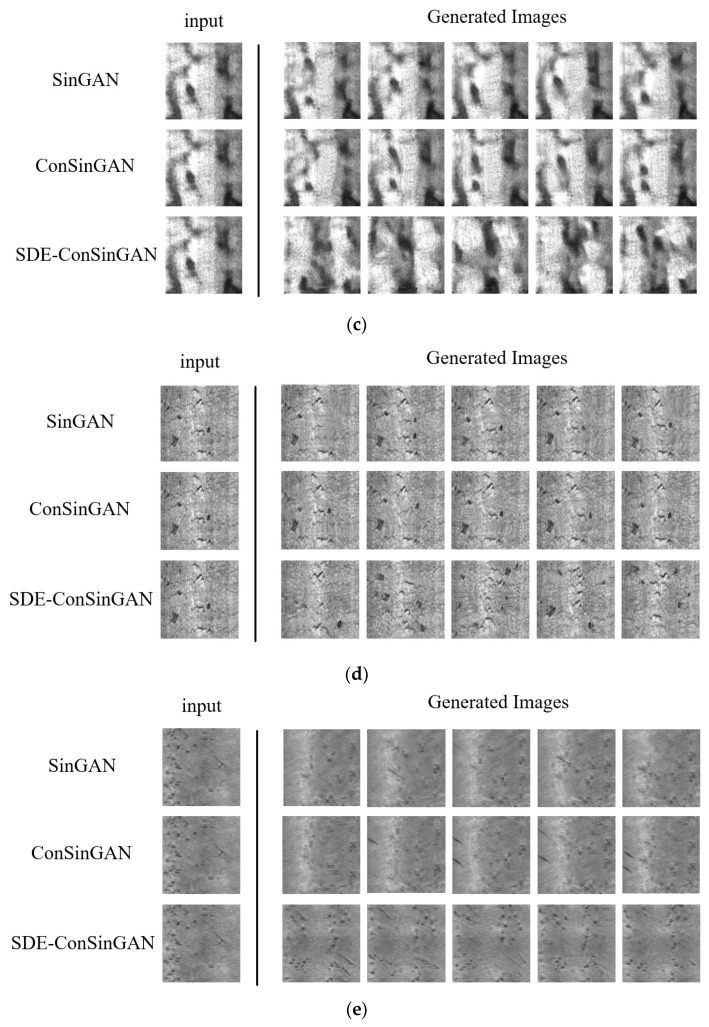

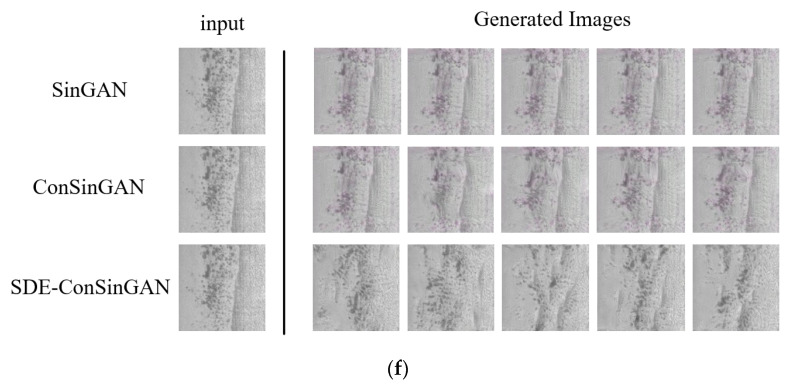
Defect image generation through various image-generation methods. (**a**) Sc. (**b**) In. (**c**) Pa. (**d**) Cr. (**e**) RS. (**f**) PS.

The proposed SDE-ConSinGAN and other single-image generative models are used to generate the six defect images in Figure 8. Some important parameters in this experiment were set as follows: the number of training stages was set as 5, the learning rate was set as 0.2, the simultaneous training stages were set as 3, the default iterations were set as 2000, the generator learning rate was set as 0.0005, and the discriminator learning rate was set as 0.0005. The training results computed by SinGAN, ConSinGAN, and SDE-ConSinGAN are shown in Figure 10. The results imply that the images generated by SDE-ConSinGAN are visually much better than those of other methods. Some defect images generated by SinGAN and ConSinGAN are meaningless and wrong because the two methods place the training focus on the low-resolution stage, coupled with high complexity and structural uncertainty. By introducing the attention mechanism, SDE-ConSinGAN pays much more attention to learning obvious image defect features and emphasizing texture. The generated defect images have less noise, better boundary effects, and more diversity. In the experiments of generating Cr, Pa, and PS defect images in Figure 10b,c,f, the images generated by SinGAN and ConSinGAN have merely slight disturbances, and most of them are invalid. The main reason lies in the fact that the two methods work well in learning the overall structure of a single image but badly in accurately and stably extracting detailed defect features.

**Figure 11:**
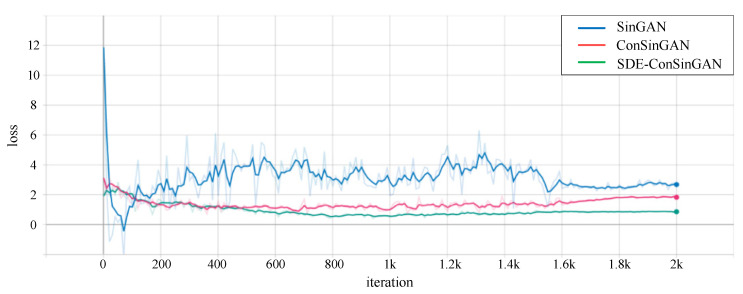
Loss comparison of three image-generation methods.

Figure 11 has displayed loss curves of the above-mentioned three methods. In Figure 11, the loss values of the generators are gradually reduced to convergence. The loss of SDE-ConSinGAN has less fluctuation and converges faster. However, although the training results of SinGAN and ConSinGAN can converge, most of their generated images do not reflect the diversity. Therefore, the loss curve cannot fully reflect the effect of strip defect image generation.

### 4.3. Image Cutting and Stitching

**Figure 12:**
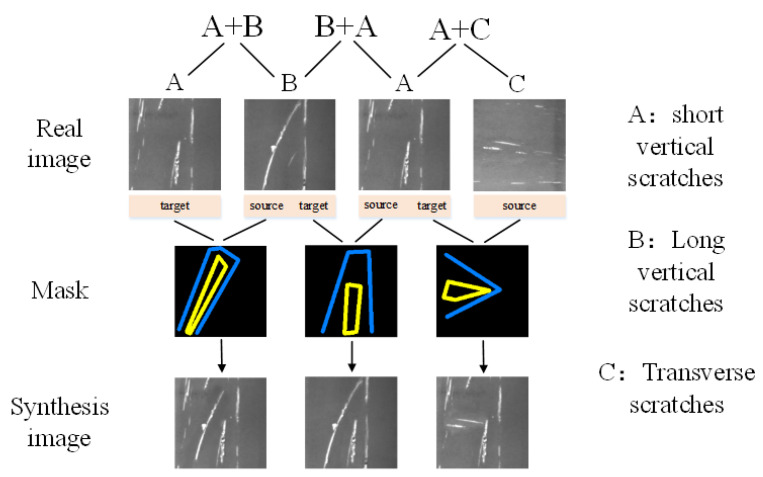
Three kinds of scratch defect image cutting and splicing.

After the model training of SDE-ConSinGAN is completed, the feature cutting and splicing operation is performed on any two original image models in order to solve the problem of creativity insufficiency of a single image. Among the two images corresponding to the same type of defect, one is selected as the source domain, and the other is regarded as the target domain. A clipping mask is created in the source domain to extract its image features, and then it is stitched into the target domain image. In this way, the new picture can have the features from two images, as is shown in Figure 12.

In Figure 12, features of three scratch defects, namely, short and long vertical scratches and transverse scratches, are cut and spliced. Only two original images can be spliced into a new image for training, and the way that source domain, target domain, and masks are selected varies greatly. Therefore, there will be more newly generated images each time an available image sample is added. The synthesized images are applied to SDE-ConSinGAN as new original samples for training, with the aim of enriching samples and obtaining more diverse images, as is exhibited in Figure 12. In this way, the SDE-ConSinGAN model can have more choices and solve the fundamental problem of less diversity of generated images.

**Figure 13:**
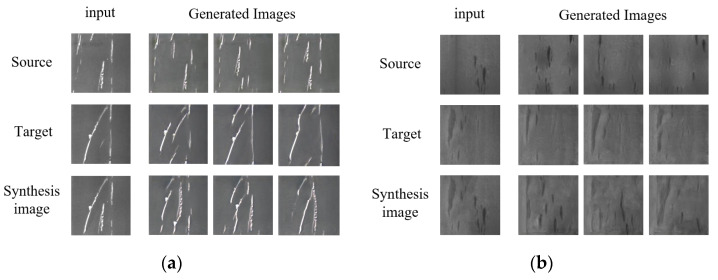

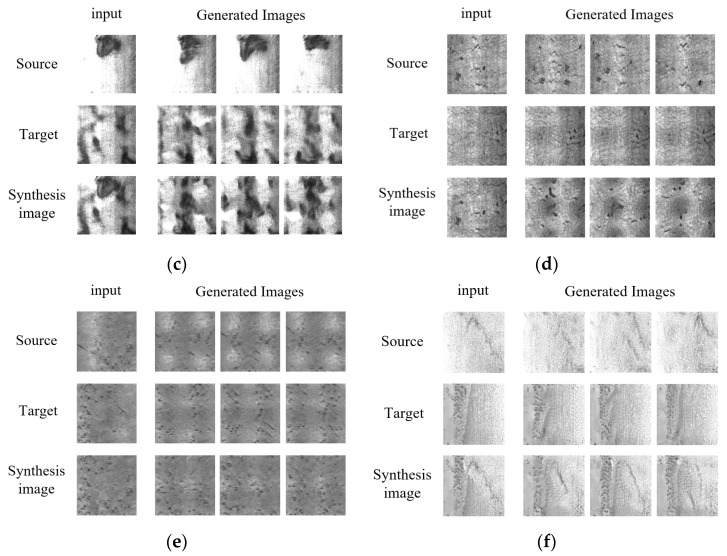
Defect image generated via image synthesis. (**a**) Sc. (**b**) In. (**c**) Pa. (**d**) Cr. (**e**) Rs. (**f**) PS.

According to the results displayed in Figure 13, it is found that it is feasible to adopt SDE-ConSinGAN for image generation and Graphcut for feature fusion. The generated images obtained through the synthetic images combined partial features of target images and original ones, thus successfully accomplishing the migration among different features from the same category.

### 4.4. Quantitative Evaluation

Since the trained loss curves and visual representation are not the best criteria to evaluate a single-image-generation model, it is necessary to adopt a quantitative evaluation method.

#### 4.4.1. Image-Generation Quality

SSIM index is used to evaluate the similarity of the generated images for each defect type in the experiments [21]. First, original images are scaled and reconstructed to the size with the same resolution as the generated images. Second, the SSIM index is obtained by calculating parameters of generated images and original images. Finally, the SSIM index of each type of image is averaged to obtain the image SSIM index. Table 2 lists the SSIM index values of six defect images through different generation methods. Each image can generate 600 images based on 10 original samples. The similarity indexes through the SSIM measurement system can be calculated through three contrast modules, namely, brightness, contrast, and structure. A larger SSIM value always indicates greater similarity of generated images to original ones. In other words, a smaller SSIM value always indicates better image generation. The experimental results fully indicate that the SSIM values among different defect types vary greatly, mainly because the image sample defects include structural and texture defects. The former means that the image defects which are in a concentrated distribution have strong structures, such as Sc, Pa, and In. The latter refers to the fact that the image defects which are in an even distribution have a strong sense of texture, such as Cr, PS, and RS.

It can be extrapolated from Table 2 that when properties of In, Pa, and Sc defects are strong in structure, there are various structure changes through a single-image-generation method that lead to a low SSIM index. On the contrary, when Cr, PS, and RS defects are weak in structure and strong in texture, there is no obvious structure change in the generated images, which leads directly to a higher SSIM index. To generate high-quality images with more diversity, the SSIM index must be kept neither too high nor too low. The images generated by SinGAN and ConSinGAN are similar in structure and have high SSIM values because they set the global image layout before learning to generate detailed features. Conversely, SDE-ConSinGAN changes the training focus, which results in an unfixed global structure, more diversity, and a low SSIM value. When the image feature is cut and spliced, its diversity is further increased, and its SSIM value is close to 0.5.

Commonly, the metric for GAN evaluation is determined via FID [18], which is used to measure the deep feature distribution deviation from the generated images to the real ones. However, like SinGAN, the SDE-ConSinGAN and Graphcut models have only one real image as an input. Single-image FID (SIFID) is an adaptation of FID to a single-image domain. It is employed to perform a comparison between the network activation statistics of generated ones and those of real ones. SIFID, an adjusted FID, is used to collect feature data of real images and generated samples [19]. Each image will generate 600 images based on 10 original samples. Table 3 displays the average scores of the 600 images.

Consistent with the FID, a small SIFID value always indicates a higher image confusion rate. When the SDE-ConSinGAN + Graphcut model is adopted, SIFID scores across all six defects are the lowest, indicating better quality and more diversity than those of other methods. In addition, the SIFID value also indicates defect complexity. A large SIFID value indicates more complexity of defect images. Therefore, Cr, PS, and RS defects have a strong texture and high complexity, while In, Pa, and Sc are converse, which is consistent with our previous results.

The training time of the defect generation methods depends mainly on network structure, training time, and data volume. Provided that experimental data, hyperparameters, and experimental environment are exactly the same, the results are displayed in Table 4 when the training stage is 6.

In Table 4, the training time of SinGAN is as long as 137 min due to its lack of a multi-stage co-training mechanism, while the training time of ConSinGAN is significantly reduced due to its simultaneous training to multiple stages. On this basis, thanks to the transition of training methods, SDE-ConSinGAN has reduced its training time by 360 s and greatly enhanced its model performance compared with those of ConSinGAN.

#### 4.4.2. Ablation Contrast Experiment

The comparison experiments are ConSinGAN, the model after replacing the image size adjustment, the model using the segmentation function of the number of iterations, and the model after adding ECA-Net. In the experiment, the total number of training stages is six. Furthermore, the comparison will be performed from three aspects, namely, the diversity of generated images, the quality of generated images, and the final results of different methods under the same number of rounds. The objects that require comparison are the sample generation results of Sc and Cr, which represent structural and textural defects, respectively.

**Figure 14:**
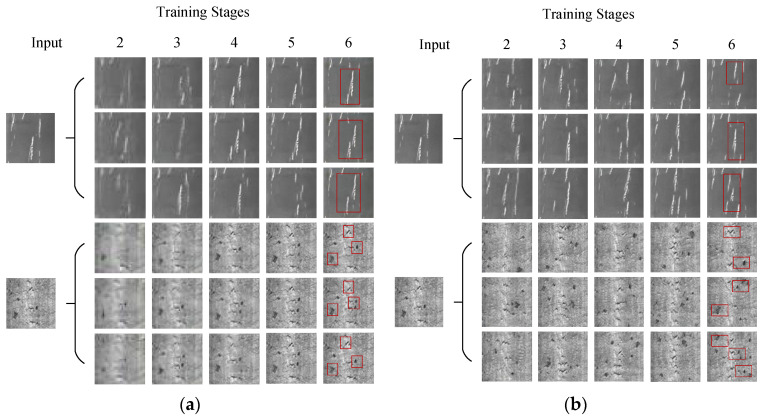

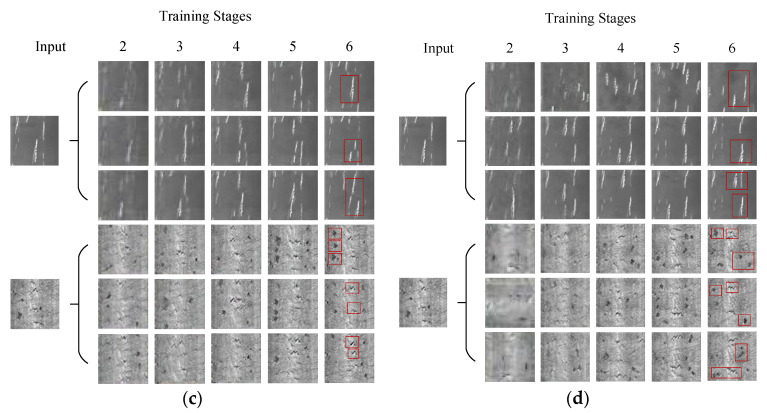
Ablation contrast experiment result. (**a**) ConSinGAN training results. (**b**) Training results after changing the means of image size change. (**c**) Training results after changing the iteration times. (**d**) Increased training results after ECA-Net.

Figure 14a shows the results generated by the model ConSinGAN. It can be seen that the higher the number of training stages is, the clearer the images are. When the training stage reaches three, the generated images have basic outlines and clear textures. After the fourth stage, the difference between the generated defect images and the original ones basically disappears, and the distribution of defects is almost the same as that of the original ones, except for some slight disturbance and change. 

As shown in Figure 14b, when the image size transformation method is changed, images become clear in the second stage because low-resolution stages have been shortened and images have learned the detailed features of defects. In the later stages, the location of defects constantly changes because images have barely learned the layout of defects. As a result, the generated images are diverse, which has successfully solved the problem of overfitting in the original model.

In the Figure 14c, after the curve function is used to control iteration times, the iteration times are reduced in stages 2–4. Consequently, the time of learning defect details is greatly shortened, and the generated samples show no obvious defect characteristics. When the iteration times are increased in stages 5~6, the characteristics of the generated samples are the same as those of the original ones. What is more, some characteristics can appear repeatedly many times. All of the above phenomena can be attributed to increased iteration times in high-resolution stages. It means that the ability of the model to learn details is greater than its ability to learn the whole layout. Compared with those original samples, the generated samples increased the density of defects.

As is exhibited in Figure 14d, after the attention mechanism of the ECA-NET channel is added, weight is added to the defect area. Consequently, defects are not filtered out via the pooling process. Moreover, the model has successfully learned defect features and neglected background noise and irrelevant features. Therefore, a variety of defects in the original samples can be well generated, which further improves the diversity of the generated samples.

Table 5 shows that when the image size transformation method is changed, the SSIM and SIFID values of defect Sc decrease by about 0.032 and 0.011, respectively; the SSIM and SIFID values of defect Cr decrease by 0.050 and 0.015, respectively. After the iteration times are changed, the SSIM and SIFID values of defect Sc decrease by about 0.03 and 0.011, respectively; the SSIM and SIFID values of defect Cr decrease by 0.05 and 0.012, respectively. After ECA is added, the SSIM and SIFID values of defect Sc decrease by 0.05 and 0.02 respectively; SSIM and SIFID values of defect Cr decrease by 0.14 and 0.013 respectively.

Combined with Table 3 and Table 4, effective conclusions can be drawn. Aimed at the problems of diversity insufficiency and overfitting of strip defect images caused by the original method, the three methods proposed in this paper have obvious effects. Each method can improve the results. Finally, the three methods are used at the same time so that the SDE-ConSinGAN model can complete the task of strip steel defect image generation well.

#### 4.4.3. Defect Sample Classification

**Figure 15:**
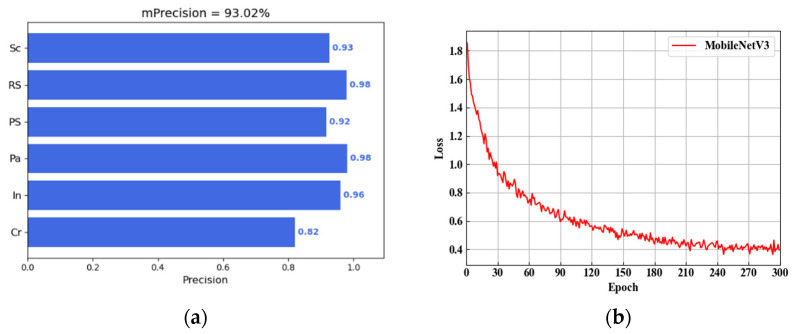
Classification precision and loss curves. (**a**) Precision. (**b**) Loss curve.

Several experiments are conducted to verify if the proposed method with a few samples is effective in defect classification improvement. First, the SDE-ConSinGAN model is employed to regenerate 800 defect samples on the basis of 10 original ones as a training dataset. Then, an additional 100 real samples are selected as a test set. As a deep-learning algorithm, MobileNetV3 uses complementary search technology in training in the search for lightweight and efficient network architecture. When it is used, it boasts such advantages as good image classification ability, a small number of parameters, and fast prediction speed. By virtue of these advantages, it can meet the requirements of industrial applications [58]. The MobileNetV3 classification network is used to train the 800 samples for 300 rounds. The experimental results and training losses are illustrated in Figure 15. In this figure, the dataset of SDE-ConSinGAN can quickly converge when the classification task is performed, and its precision rate reaches 93.02%. The results indicate that the generated samples are effective and reliable through SDE-ConSinGAN.

Then, SinGAN and ConSinGAN are adopted to regenerate 800 samples on the basis of the same training parameters as a training dataset for subsequent classification. Each method is tested five times, and their calculated results are shown in Table 6. Predicted time refers to the average time of predicting 100 images in the testing set. The data in the table, namely accuracy, precision, recall, and F1 scores, are the average values of each type of image in the test after five experiments.

In Table 6, when SDE-ConSinGAN and Graphcut is employed, the error rate of the defect identification model is lower than that of others, and its training process is more stable. Due to the small number and poor diversity of defect images, training datasets that were increased after SinGAN and ConSinGAN are used for sample enrichment. However, as semantic features are limited by single-image-generation models, generated sample diversity does not increase significantly, making it difficult to stably train the model for defect classification. Fortunately, SDE-ConSinGAN can not only enrich defect images but also generate more diversified images. In other words, it has numerous advantages, such as being free from generating invalid images, improving the overall dataset quality, and being able to train a better classification network. Compared with the other methods, the accuracy rate, precision rate, F1 score, and other indicators are improved by about 5%. Combined with Graphcut, it provides more raw samples for the generation of more reliable and high-quality images, thus further improving the diversity of defect image generation. However, in spite of this, the classification accuracy of samples generated via SDE-ConSinGAN is similar to that of real NEU defect images. Actually, we do not know how many real samples to train to generate the model to the best effect, and we chose 10 samples arbitrarily. However, when the 10 original defect images that were used to expand the samples were increased to 15, the classification result reached 97.5%. When the original defect images were increased to 20, the classification accuracy was close to 99%, which was far beyond the classification accuracy of using real NEU samples. Although we increased the number of samples from 10 to 20, compared with other generation models, SDE-ConSinGAN is still a generation model available with fewer samples. In a word, the proposed method SDE-ConSinGAN, which requires fewer real samples and has higher image quality and diversity, can accurately and comprehensively reflect real features of the defect dataset.

## 5. Discussion

In this paper, a novel method named SDE-ConSinGAN is put forward, which is a GAN based on training a single image for strip steel surface defects. It aims to solve the problem that such algorithms as strip steel defect detection or classification based on deep learning under few samples cannot be trained or are not accurate due to sample insufficiency. We perform data augmentation using SDE-ConSinGAN and other single-image-generation algorithms and train a defect classification model using the augmented dataset. The research results are as follows:

(1) According to the characteristics of the industrial defect dataset, the model places training focus on high image resolution. The image global structure is first roughly learned, and then the texture and style details are given top priority for learning. Meanwhile, through sample feature cutting and splicing, diversity number and feature types can be chosen according to the actual condition of image generation.
(2) In terms of training speed, iteration times are changed in each stage, which can shorten training time and improve model performance. Adding an attention mechanism to the generator allows the training model to focus on feature learning and to neglect background noise.
(3) Based on the analysis of Figure 14, we can conclude that SDE-ConSinGAN endows the network with higher convergence speed and accuracy. Since SDE-ConSinGAN endows more high-resolution training stages, the model is forced to focus on learning the detailed features of the image rather than the global features during the training process, and the generated samples reduce the occurrence of repeated features. On the other hand, modifying the first item of the loss function ensures the stability of the model in the early stage of training. The number of iterations in the high-resolution stage is increased by using dynamically adjusted iterations, which not only speeds up the training speed but also conforms to the global characteristics of model learning. Based on the above adjustments, the problem that ConSinGAN cannot be applied to defective samples is effectively solved. By comprehensively comparing these methods in Figure 13, we noticed that SDE-ConSinGAN has excellent data enhancement capabilities for different types of strip steel defect samples. In particular, when the features are not prominent or obvious, SDE-ConSinGAN performs steadily better.
(4) In Table 2, Table 3 and Table 4, the proposed method achieves lower SSIM and SIFID and faster training speed. However, the performance of other methods is not very satisfactory. The results show that the proposed SDE-ConSinGAN can be trained on a single image. In addition, the training speed is faster, and the average SSIM index of the generated samples is 0.600, which is 0.163 lower than that of ConSinGAN. The average SIFID index is 0.086, which is 0.052 lower than that of ConSinGAN. In Figure 15 and Table 6, the accuracy rate of the defect classification task with the dataset generated by SDE-ConSinGAN reaches 93.02%, which is higher than that of SinGAN and ConSinGAN. It can be concluded that the proposed method can be applied to the generation of strip surface defect samples. The intrinsic reason is that the structure of the strip image has great uncertainty. When the generator learns strip defect categories, it mainly learns from the details such as color and texture. Therefore, it is possible to stably learn and generate similar samples when the gray scale changes obviously have large-scale defect changes and most small features. The networks of other methods do not make weight trade-offs for learning features, and these networks do not increase attention mechanisms. Thus, the sample data obtained via SDE-ConSinGAN have higher diversity and reliability, which proves the feasibility in the field of strip defect image generation.
(5) Compared with ConSinGAN, SDE-ConSinGAN changes the training mode of images. Therefore, in addition to the generation of strip steel defect samples, SDE-ConSinGAN can also be applied to other industrial defects. In a broad sense, SDE-ConSinGAN can be applied to the generation of samples that are insensitive to global features. The shortcoming of this paper is that SDE-ConSinGAN is not effective in processing large block defects, and the efficiency of Graphcut is low, which can be optimized appropriately. Meanwhile, in the follow-up research, the ability of SDE-ConSinGAN to extract image features will be improved, such as by adding edge feature-enhancement modules.

## Figures and Tables

**Table 1:** Comparison of image size-adjustment effects of two methods.

	Stage	1	2	3	4	5	6
Model							
ConSinGAN		42 × 42	50 × 50	62 × 62	85 × 85	146 × 146	200 × 200
SDE-ConSinGAN		55 × 55	89 × 89	124 × 124	151 × 151	170 × 170	200 × 200

**Table 2:** SSIM indices of images generated by several models.

	Defect Type	Cr	In	Pa	PS	RS	Sc
Model							
SinGAN		0.835	0.744	0.718	0.812	0.857	0.692
ConSinGAN		0.880	0.790	0.691	0.803	0.803	0.613
SDE-ConSinGAN		0.713	0.623	0.618	0.648	0.726	0.544
SDE-ConSinGAN + Graphcut (ours)		0.654	0.559	0.576	0.607	0.684	0.521

**Table 3:** SIFID scores of images generated via various image-generation methods.

	Defect Type	Cr	In	Pa	PS	RS	Sc
Model							
SinGAN		0.153	0.142	0.144	0.167	0.164	0.137
ConSinGAN		0.126	0.115	0.135	0.162	0.166	0.128
SDE-ConSinGAN		0.0981	0.0843	0.1013	0.1276	1.0025	0.1069
SDE-ConSinGAN + Graphcut (ours)		0.0844	0.0732	0.0947	0.0992	0.0816	0.0850

**Table 4:** Time comparison of different image-generation methods.

Model	Training Stages	Epoch (Total)	Training Time (min)
SinGAN	6	12,000	137.469
ConSinGAN	6	12,000	33.518
SDE-ConSinGAN	6	10,829	27.445
SDE-ConSinGAN + Graphcut (ours)	6	10,829	26.876

**Table 5:** Comparison of SSIM and SIFID values in ablation experiments.

Model	SSIM (Sc)	SSIM (Cr)	SIFID (Sc)	SIFID (Cr)
ConSinGAN	0.611	0.880	0.128	0.126
+Image size adjustment	0.583	0.832	0.117	0.114
+Iteration times variation function	0.585	0.817	0.114	0.110
+ECA-Net	0.565	0.744	0.107	0.113

**Table 6:** Defect classification performance of different image-generation networks.

Model	Predicted Time/ms	Accuracy/%	Precision/%	Recall/%	F1
NEU (real)	250	91.56	91.89	92.03	91.95
SinGAN	278	85.16	86.49	87.05	86.77
ConSinGAN	291	88.73	88.51	88.96	88.73
SDE-ConSinGAN	305	92.87	93.06	92.67	92.86
SDE-ConSinGAN + Graphcut (ours)	308	94.67	94.36	94.24	94.30

## Data Availability

The data presented in this study are available in Ref. [57].
